# FN1 is a prognostic biomarker and correlated with immune infiltrates in gastric cancers

**DOI:** 10.3389/fonc.2022.918719

**Published:** 2022-08-23

**Authors:** Han Wang, Junchang Zhang, Huan Li, Hong Yu, Songyao Chen, Shuhao Liu, Changhua Zhang, Yulong He

**Affiliations:** ^1^ Department of Center for Digestive Disease, The Seventh Affiliated Hospital of Sun Yat-sen University, Shenzhen, China; ^2^ Department of Gastrointestinal Surgery, The First Affiliated Hospital of Sun Yat-sen University, Guangzhou, China

**Keywords:** gastric cancer, FN1, tumor-associated macrophages, prognosis, tumor immune microenvironment

## Abstract

Fibronectin 1 (FN1) is a glycoprotein found throughout the extracellular matrix that has a role in the onset and progression of cancer. However, its immune relationship with gastric cancer is still unclear. FN1 was systematically reviewed by Gene Expression Profiling Interactive Analysis (GEPIA), Linked Omics, Tumor IMmune Estimation Resource (TIMER), and Kaplan–Meier (KM) plotter analysis. The TIMER, GEPIA, TISIDB, and cBioPortal databases investigated the association of FN1 with tumor immune infiltration and validated using immunohistochemistry. We discovered that tumor tissue expresses FN1 at a higher level than neighboring tissue, and those genes coexpressed with FN1 have a poor prognosis. At the same time, we discovered that increased FN1 expression was related to immunological infiltration, particularly macrophage infiltration. Using immunohistochemistry, we discovered that FN1 expression was tightly connected to M2 macrophages. It can be concluded that FN1 can affect the immunological microenvironment and is a prognostic marker in gastric cancer.

## Introduction

Gastric cancer (GC) is the fifth most frequent cancer and the fourth main cause of mortality worldwide (1); however, the survival rate of end-stage GC is less than 1 year (2). Because most GCs are found at an advanced stage, prevention and treatment of GC remain a top priority. Radical surgery, with or without adjuvant chemotherapy, is the only approach to treat localized GC. Chemotherapy, on the other hand, is the primary therapeutic option for metastatic GC (3). Immunotherapies, such as immune checkpoint inhibitors, have recently emerged for the treatment of patients with advanced GC, but in most patients, they are not promising (4). As a result, elucidating the immunophenotype of tumor–immune interactions and identifying novel immune-related therapeutic targets in GC are urgently needed.

The tumor microenvironment plays an important role in tumor development, invasion, and metastasis (5). Cancer cells can interact with their extracellular matrix (ECM) during proliferation and migration. Fibronectin 1 (FN1) is an important ECM glycoprotein with several alternatively spliced variants (6). It has been shown to be involved in cell proliferation and migration and through integrin transmembrane receptors in ECM changes that occur during physiological and pathological processes (7, 8). FN1 promotes the migration and invasion of papillary thyroid cancer (9), colon cancer cells (10), clear cell renal cell carcinoma (11), etc. However, the possible role of FN1 in regulating tumor immunity remains unclear.

In this study, FN1 expression and its relationship with the prognosis of GC patients were investigated using multiple databases including Gene Expression Profiling Interactive Analysis (GEPIA), Link Omics Database, and Kaplan–Meier (KM) plotter datasets. In addition, Tumor IMmune Estimation Resource (TIMER), cBioPortal, and immunohistochemical studies were performed to explore the relationship of FN1 and different immune-related cells in tumor microenvironments. This study sheds light on the critical role of FN1 in GC and possible links and mechanisms by which FN1 regulates tumor-infiltrating immune cells.

## Methods

### TIMER database analysis

TIMER2.0^1^ is a web-based interactive platform for the systematic analysis of immune infiltration in various malignancies. TIMER2.0 applies six advanced algorithms to more rigorously assess levels of tumor-infiltrating lymphocytes (TILs) from The Cancer Genome Atlas (TCGA) or tumor-related data. We investigated the expression of FN1 in various malignancies and the relationship between FN1 and TIL expression through gene modules. Furthermore, the link between FN1 expression and gene signatures of TILs, including CD8+/CD4+ T cells, tumor-associated macrophages (TAMs), M1 macrophages, M2 macrophages, T cells, and related subtypes has been analyzed. An expression scatter plot between Spearman’s correlation and estimated statistical significance for a pair of genes for GC was constructed using the correlation module. The levels of gene expression are represented as log2 RSEM.

### Kaplan–Meier plotter [gastric cancer]

The KM plotter^2^ included 1,065 GC samples with a mean follow-up of 33 months. It could assess the survival prognosis of related genes and draw the corresponding survival curve. The KM plotter evaluates the prognostic significance of FN1 in GC, including overall survival (OS), first progression (FP), and postprogression survival (PPS). Hazard ratios (HRs) with 95% confidence intervals and log-rank p-values were also estimated. Statistical significance was defined as p < 0.05.

### Linked omics database analysis

Linked Omics Database^3^ is a public portal that includes multi-omics data from all 32 TCGA cancer types and 10 Clinical Proteomics Tumor Analysis Consortium (CPTAC) cancer cohorts, which provides biologists and clinicians with a unique platform to access, analyze, and compare multi-omics data within and across tumor types. The differentially expressed genes related to FN1 were screened from TCGA stomach adenocarcinoma (STAD) cohort by the LinkFinder module in the database, and the Pearson correlation coefficient was used to test the results, which were shown as volcano plots and heat maps, respectively. Function module analysis of Gene Ontology biological process (GO_BP), Kyoto Encyclopedia of Genes and Genomes (KEGG) pathways by the gene set enrichment analysis (GSEA) in the LinkInterpreter module.

### Gene expression profiling interactive analysis

The GEPIA^4^ online database is a comprehensive platform for analyzing the RNA sequencing expression data of 9,736 tumors and 8,587 normal samples from TCGA and the Genotype-Tissue Expression (GTEx) projects. We assessed FN1 levels in GC samples and healthy samples using the GEPIA data source *via* the DIY expression page.

### cBioPortal for cancer genomics

The cBioPortal^5^ for Cancer Genomics was originally developed at Memorial Sloan Kettering Cancer Center (MSK). The public cBioPortal site is hosted by the Center for Molecular Oncology at MSK. We utilized the cBioPortal to visualize and compare gene changes.

### Immunohistochemistry

This study was performed on 150 paraffin-embedded GC specimens from the Department of Pathology, The First Affiliated Hospital of Sun Yat-sen University. Immunohistochemistry (IHC) was performed to study the expression of FN1 and CD163 to determine the relationship between FN1 expression and CD163. Anti-FN1 (1:500; Proteintech, 66042-1-Ig) and anti-CD163 (1:400; Cell Signaling Technology, #93498) were used for IHC staining. Staining intensities were categorized as follows: negative (-), weak (+), moderate (++), and strong (+++).

### Statistical analysis

The level RNA sequencing (RNA-seq) data of STAD, named TCGA-STAD, were obtained from TCGA database^6^. For additional analysis, counts and fragments per kilobase of transcript per million mapped reads (FPKMs) were retrieved, as well as clinical information from matched patients. KM plots were performed to construct survival curves. For KM plots, GEPIA, and TISIDB, HR and p-values were described using the log-rank test. To investigate the FN1 expression, immune infiltration levels, and immunological checkpoints, Spearman correlation coefficients were obtained. All analyses were carried out with the help of GraphPad Prism (version 8.00), R (version 3.6.3), or SPSS statistics (version 26). Statistical significance was defined as p < 0.05.

## Results

### Fibronectin 1 is highly expressed and is associated with poor prognosis in gastric cancer

**Figure 1:**
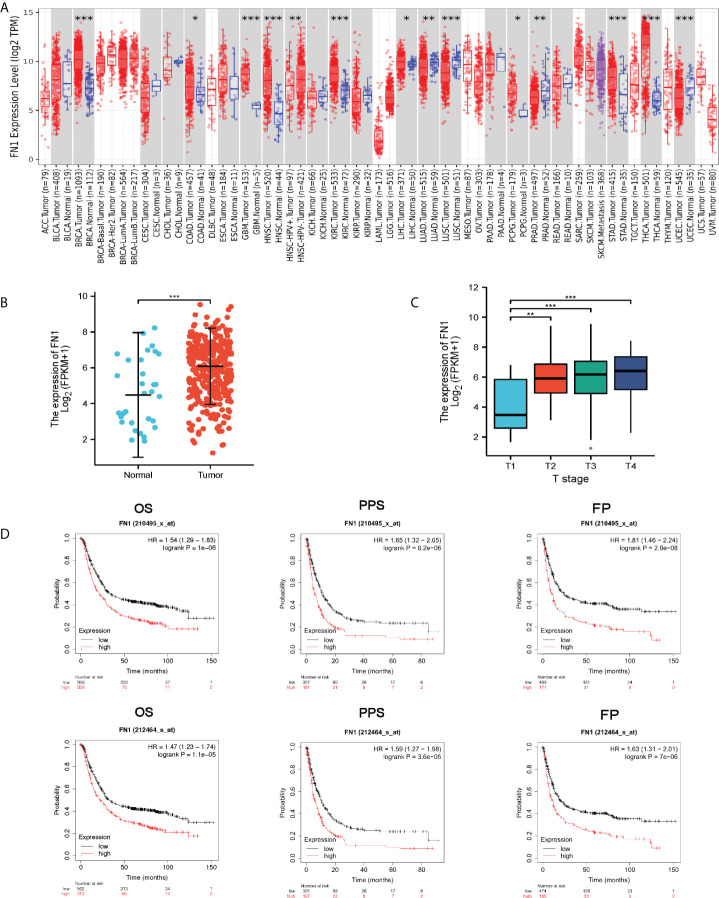
FN1 expression levels in different types of human cancers. **(A)** Human FN1 expression levels in different tumor types from TCGA database were determined by TIMER (*p < 0.05, **p < 0.01, ***p < 0.001). **(B)** FN1 mRNA expression in STAD tissues and normal tissues (N = 32; T = 375). **(C)** Association of FN1 expression with T stage. **(D)** OS, FP, and PPS survival curves of gastric cancer. OS, overall survival; FP, first progression; DFS, Disease free survival.

Maintenance of proliferative signaling, stimulation of angiogenesis, encouragement of invasion and metastasis, regulation of growth inhibitory activity, and modulation of antitumor immunity are all crucial biological qualities necessary for cancer. FN1 also possesses these capabilities (12). Firstly, we analyzed FN1 transcription levels in different human tumors (**Figure 1A**). FN1 mRNA levels is higher in GC tissues rather than normal gastric tissues in TCGA (**Figure 1B**). Next, we explored the association of FN1 expression with T stage (**Figure 1C**). We evaluated the prognostic in GC using GEPIA with TCGA data and in the KM plotter database with Gene Expression Omnibus (GEO) data. It indicated that higher expression of FN1 was significantly related to shorter OS in the GC cohort 210495-x-at (OS: HR = 1.54, p < 0.001) and 212464-s-at (OS: HR = 1.47, p < 0.001) (**Figure 1D**). The findings show that FN1 is overexpressed in GC and is linked to a poor prognosis.

### Diagnostic value of fibronectin 1 mRNA expression in gastric cancer

**Figure 2:**
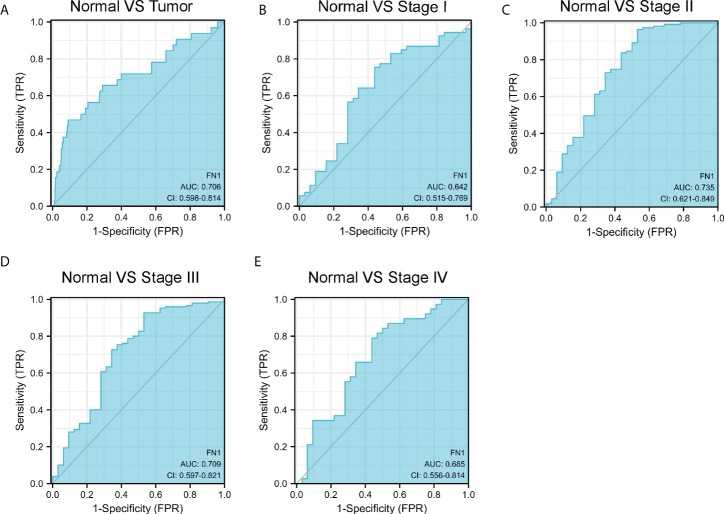
ROC curve of FN1 mRNA expression in STAD cohort. **(A)** ROC curve of FN1 mRNA expression in normal and tumor. **(B–E)** Subgroup analysis for stages I, II, III, and IV, respectively.

Many pathological diseases, including rheumatoid arthritis, infection, and cancer, have been tied to FN1 (12–14). Meanwhile, FN1 has been linked to cancer patients’ prognosis and therapy responsiveness (15). To determine if FN1 has diagnostic value, we used a receiver operating characteristic (ROC) curve to assess the diagnostic value of FN1 mRNA expression. The results revealed that the area under the curve (AUC) of FN1 was 0.706 (**Figure 2A**). We also analyzed the diagnostic capability of FN1 mRNA expression in different stages, and the results showed a similar diagnostic value with AUC values of 0.642, 0.735, 0.709, and 0.685 for stages I, II, III, and IV, respectively (**Figures 2B–E**).

### Fibronectin 1 coexpression network in stomach adenocarcinoma

**Figure 3:**
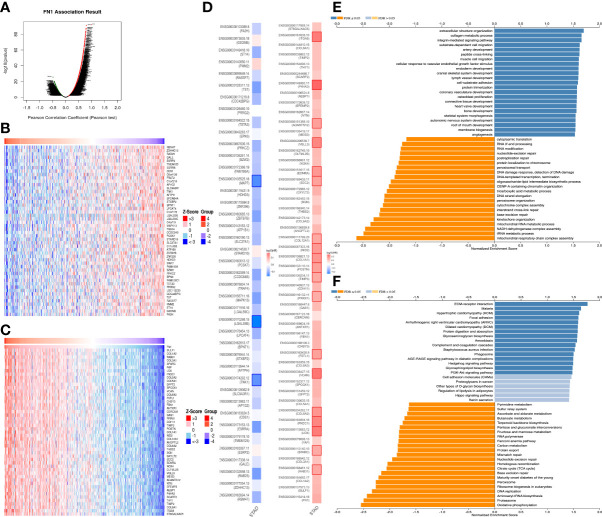
The coexpression genes with FN1 in STAD from the LinkedOmics database. **(A)** The whole significantly associated genes with FN1 distinguished by Pearson test in the STAD cohort. **(B, C)** Top 50 genes positively and negatively related to FN1 in STAD showed, respectively, by heat maps. Red represents positively linked genes, and blue represents negatively linked genes. **(D)** Survival map of the top 50 genes positively and negatively associated with FN1 in STAD. **(E, F)** KEGG pathways and GO annotations of FN1 in the STAD cohort.

For gaining the knowledge of FN1 biological function in STAD, the Link Finder module in the Linked Omics web portal was deployed to check the coexpression pattern of FN1 in TCGA-STAD. As is plotted in **Figure 3A**, it showed that 10,073 genes (dark red dots) positively correlated with FN1, and 10,152 genes (dark green dots) negatively correlated with FN1. **Figures 3B, C** showed the heat maps of the top 50 genes positively and negatively associated with FN1, respectively. Unsurprisingly, it is proven that the top 50 positively correlated genes highly owned the probability of becoming high-risk markers in STAD, of which all genes had an unfavorable protective HR. In contrast, there were 34 of the top 50 genes with HR in the top 50 negatively significant genes (**Figure 3D**).

KEGG pathway analysis indicated enrichment in ECM-receptor, Malaria, Hypertrophic cardiomyopathy, Focal adhesion, Arrhythmogenic right ventricular cardiomyopathy, Dilated cardiomyopathy, Protein digestion and absorption, Glycosaminoglycan biosynthesis, etc. (**Figure 3E**). GO term annotation showed that the coexpressed genes of FN1 join mainly in extracellular structure organization, collagen metabolic process, integrin-mediated signaling pathway, substrate-dependent cell migration, artery development, peptide cross-linking, muscle cell migration, etc. (**Figure 3F**). These results show a wide influence of FN1 expression network on the prognosis and immune activation in STAD.

### Gene set enrichment analysis identifies a fibronectin 1-related signaling pathway

**Figure 4:**
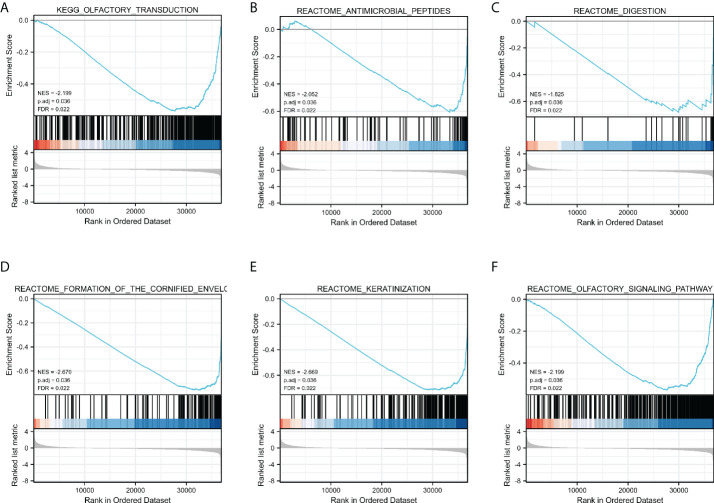
Enrichment plots by GSEA. Transduction **(A)**, Antimicrobial peptides **(B)**, digestion **(C)**, Formation of the cornified envelope **(D)**, keratinization **(E)**, and Olfactory **(F)** Signaling Pathway of DEGs altered by FN1 were involved in patients with GC from TCGA database.

GSEA was used to determine which signaling pathways were active in GC. The results showed that transduction, antimicrobial peptides, digestion, Formation of the cornified envelope, keratinization, and Olfactory Signaling Pathway were differentially enriched in the positively correlated FN1 mRNA expression phenotype (**Figures 4A–F**).

### Correlation between fibronectin 1 expression and immune infiltration

**Figure 5:**
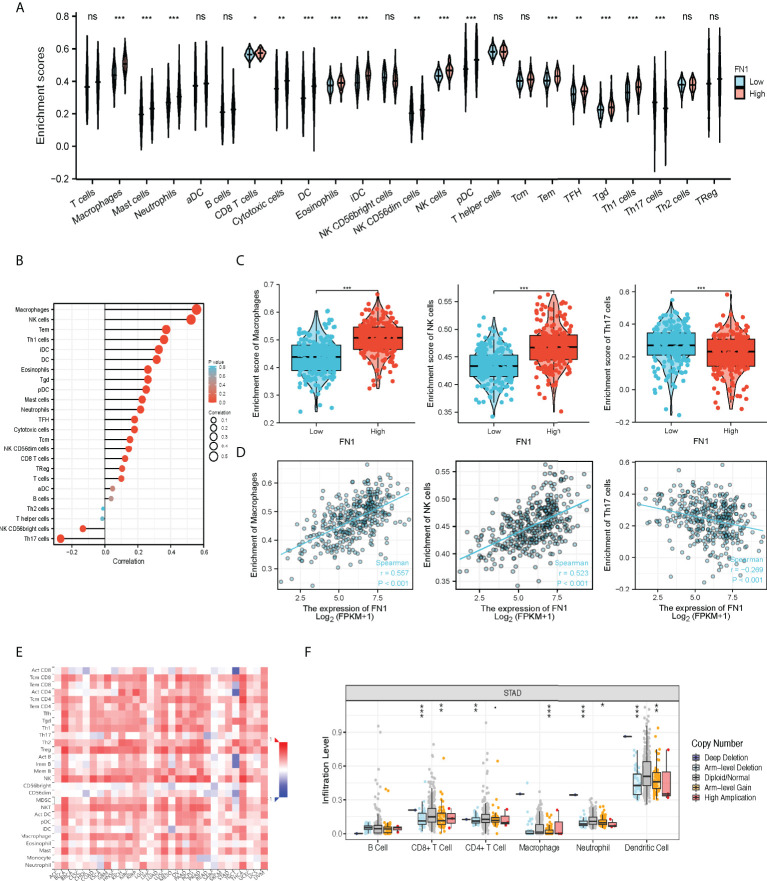
The correlation of FN1 expression with immune infiltration level in STAD. **(A)** CIBERSORT was employed to assess the difference of immune cells between FN1 high or low tumors. **(B)** The correlation between the infiltration of immune cells and the expression of FN1. **(C, D)** FN1 expression significantly positively correlates with infiltrating levels of Macrophage cells and NK cells but negatively correlates with infiltrating levels of Th17 cells. **(E)** Correlation between the expression of FN1 and the abundance of TILs in gastric cancer available at the TISIDB database. **(F)** Somatic copy number variation (CNV) of FN1 at the level of immune infiltration in gastric cancer. *p < 0.05, **p < 0.01, ***p < 0.001, nc p > 0.05.

Excessive ECM deposition occurs in the infiltration of many immune cells around tumor cells, yet they are unable to effectively enter the tumor tissue and remain in the neighboring stroma to aid immunological escape (16–18). The Spearman correlation was used to examine the relationship between the expression level transcripts per kilobase million (TPM) of FN1 and the measured level of immune cell infiltration. The expression of FN1 was negatively correlated with the abundance of T helper cells 17 (Th17) cells and natural killer (NK) CD56 bright cells and positively correlated with the abundance of macrophages and NK cells (**Figures 5A–D**, p < 0.001). TAMs have an essential role in the tumor ECM microenvironment, influencing its proliferation and modification. FN1 was found to be extremely upregulated in human ovarian cancer (19) and colorectal cancer models (20). By TISIDB, we discovered that FN1 was related to immune cells, especially mast, macrophages, Natural killer T cells (NKT, regulatory T cells) (Treg), etc. (**Figure 5E**). We compared the tumor infiltration immune cells with different copy number variations including deep deletion, diploid/normal, arm-level deletion, arm-level gain, and high amplification for FN1 in STAD. The results showed that for STAD, arm-level gain of FN1 was related to CD8+ T cells, CD4+ T-cell infiltration, macrophages, neutrophils, and dendritic cells (DCs) (**Figure 5F**, **Supplementary Table S1**).

**Figure 6:**
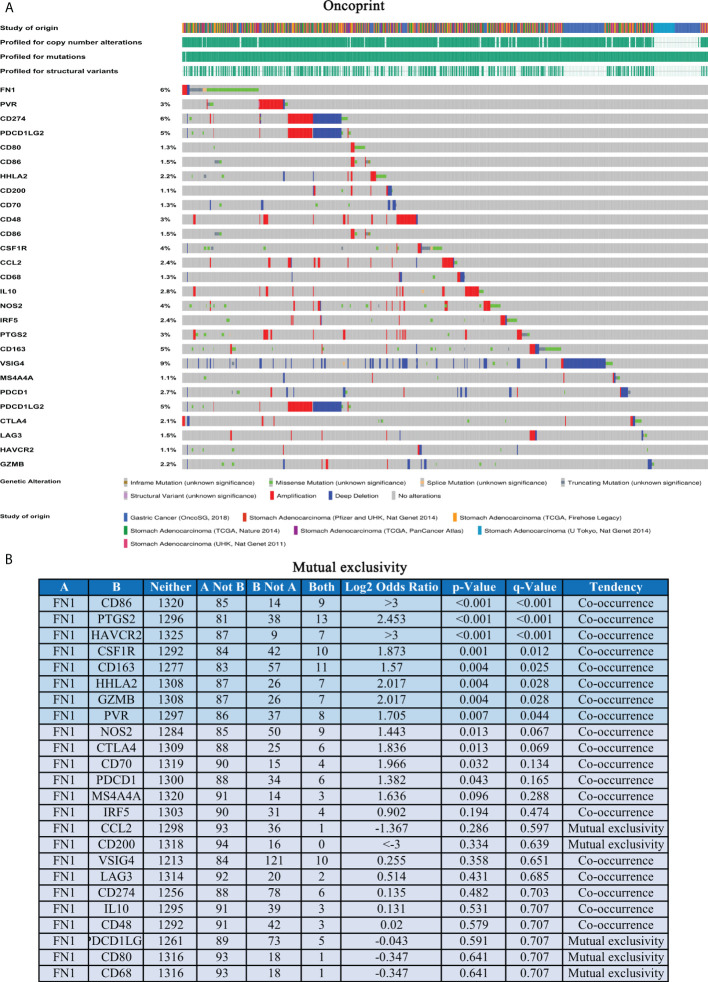
FN1 with immune checkpoints and gene markers of macrophages in STAD. **(A)** Landscape of FN1 and immune checkpoint alteration and gene markers of macrophages in STAD. **(B)** Mutual-exclusivity analysis between FN1 and multiple-immune checkpoints and gene markers of macrophage in STAD. The altered relationship between FN1 and each immune checkpoint and gene marker of macrophage. The detailed log2 odds ratio, p-value, Q-value, tendency, and significance were individually presented in each panel. Q-value <0.05 was considered to be statistically significant (indicated as yes, and others as no).

The above results turned out that FN1 is closely associated with macrophages, and genomic studies have indicated that FN1 is involved in the changes of GC immune checkpoints and macrophage markers. The overall picture of FN1 and immune checkpoint and macrophage marker alterations in GC was compactly visualized, including fusions, amplifications, deep deletions, truncations, and missense mutations (**Figure 6A**). Detailed relationships between FN1 and representative immune checkpoint and macrophage markers are shown in **Figure 6B**. Of note, the FN1 alteration showed a statistically significant co-occurrence rather than mutual exclusivity with extensive immune checkpoints, such as CD86, HAVCR2, HHLA2, and it also co-occurrence with macrophages marker, such as PTGS2, CSF1R, CD163. These results revealed the strong interaction between FN1-immune checkpoint and macrophages.

### Fibronectin 1 expression correlates with macrophage-related markers and poor prognosis in gastric cancer

**Figure 7:**
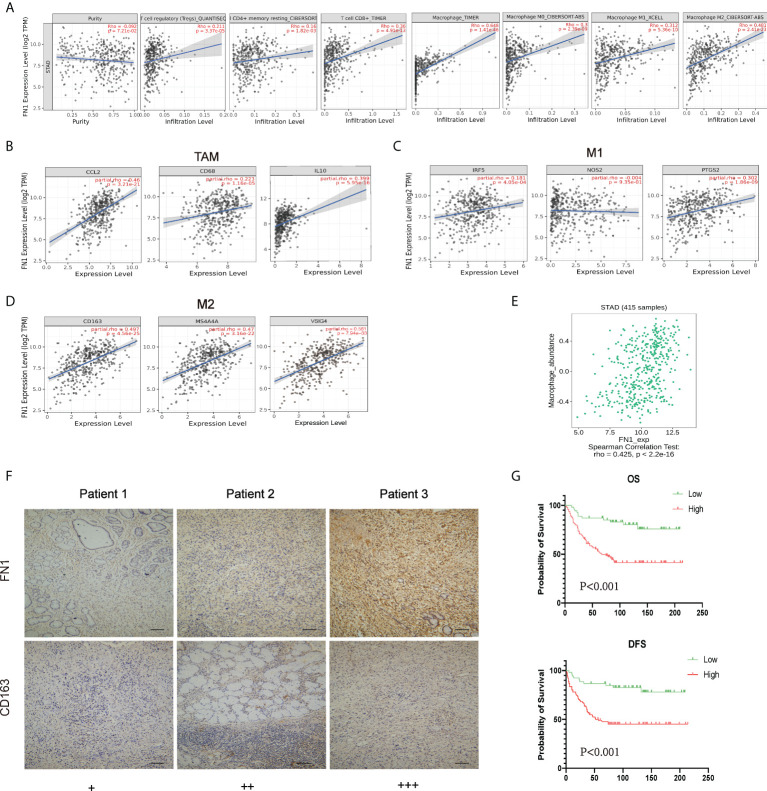
Correlation analysis between FN1 and related genes and markers of macrophages. **(A)** FN1 expression is negatively related to tumor purity and has significant positive correlations with infiltrating levels of Tregs, CD4+ T cells, CD8+ T cells, and macrophages in STAD. **(B–D)** Scatterplots of correlations between FN1 expression and gene markers of macrophages, TAMs **(B)**, and M1 **(C)** and M2 macrophages **(D)** in STAD. **(E)** Correlation between FN1 expression and Macrophage in gastric cancer available at the TISIDB database. Color images are available online (n = 415). **(F)** Correlation between FN1 expression and CD163 in gastric cancer. Gastric cancer tissue was quantitated by scoring staining intensity, including negative (–) and weak (+) staining, moderate (++) and strong (+ + +) staining, respectively. Scale bar = 100 μm. **(G)** OS and DFS analysis of FN1 in STAD.

FN1 expression is adversely correlated with the purity of STAD (rho = -0.092, p < 7.21e^−2^). High-expression level of FN1 was correlated with the infiltrating degree of Tregs (rho = 0.211), CD4+ T cell (rho = 0.16), CD8+ T cell (rho = 0.36), macrophage (rho = 0.648), macrophage M0 (rho = 0.3), macrophage M1 (rho = 0.312), and macrophage M2 (rho = 0.481) (**Figure 7A**). Besides CD4+ T cell, all the p-values were far less than 0.001. These results indicated that FN1 plays a key function in immune infiltration of GC.

Obviously, FN1 had a significant association with the majority of marker sets of TAMs, M1 macrophages, and M2 macrophages in STAD. Specifically, this study implicated that chemokine ligand (CCL)-2 and Interleukin 10 (IL10) of TAM markers are all strongly correlated with FN1 in STAD, as well as Interferon Regulatory Factor 5 (IRF5), Prostaglandin-Endoperoxide Synthase 2 (PTGS2) of M1 phenotype, and CD163, V-Set And Immunoglobulin Domain Containing 4 (VSIG4), Membrane Spanning 4-Domains A4A (MS4A4A) of M2 phenotype (p < 0.001; **Figures 7B–D**, **Table 1**). By TISIDB, we also found that FN1 was positively correlated with macrophage (**Figure 7E**). Therefore, we conducted an immunohistochemical method to determine the association between FN1 and CD163 expression, and the results revealed that the infiltration of CD163 was also increased when FN1 was highly expressed (**Figure 7F**). Simultaneously, we investigated the connection between FN1 expression and clinicopathological characteristics in GC patients (**Supplementary Table S2**). And this study showed that increased FN1 expression was linked with a worse OS and DFS in the GC (**Figure 7G**). Furthermore, univariate and multivariate Cox risk regression analyses of OS and disease-free survival of patients with GC revealed that FN1 was one of the independent risk factors for GC (**Table 2**, **Supplementary Table S3**). As a result, we concluded that FN1 is a prognostic biomarker in GC and is accompanied with immune infiltrates.

**Table 1:** Correlation analysis between FN1 and related genes and markers of macrophage in TIMER2.0.

Description	Gene markers	STAD				
		None			Purity	
		Cor	p		Cor	p
TAM	CCL2	0.463	***		0.46	***
	CD68	0.229	***		0.223	***
	IL10	0.383	***		0.399	***
M1 Macrophage	INOS (NOS2)	-0.001	0.981		-0.004	0.935
	IRF5	0.167	***		0.181	***
	COX2(PTGS2)	0.293	***		0.302	***
M2 Macrophage	CD163	0.505	***		0.497	***
	VSIG4	0.553	***		0.561	***
	MS4A4A	0.463	***		0.47	***

STAD, stomach adenocarcinoma; TAM, tumor-associated macrophage; Cor, R value of Spearman’s correlation; None, correlation without adjustment; Purity, correlation adjusted by purity; CCL2, C-C Motif Chemokine Ligand 2; CD68, CD68 Molecule; IL10, Interleukin 10; INOS, Inducible nitric oxide synthase; IRF5, Interferon Regulatory Factor 5; COX2, Regulation of cyclooxygenase 2; CD163, CD163 Molecule; VSIG4, V-Set And Immunoglobulin Domain Containing 4; MS4A4A, Membrane Spanning 4-Domains A4A.

**Table 2:** Univariate and multivariate Cox risk regression analyses of overall survival in patients with gastric cancer.

Variable	Univariate			Multivariate		
	HR	95% CI	p	HR	95% CI	p
Age (years)						
≥60 vs. <60	1.563	0.951-2.569	0.078			
Gender						
Men vs. Women	1.557	0.957-2.533	0.075			
Tumor size						
>5 cm vs. ≤5 cm	2.862	1.746-4.693	**<0.001**	1.798	1.009-3.205	**0.008**
Borrmann type						
III–IV vs. I–II	2.488	1.695-3.652	**<0.001**			
Differentiation						
Poor vs. Well+ moderate	2.685	0.372-19.362	0.327			
poor vs. moderate						
T3–T4 vs. T1–T2	5.070	2.186-11.76	**<0.001**			
Lymph node metastasis						
N+ vs. N0	2.860	1.673-4.887	**<0.001**			
LVI						
Present vs. none	2.134	0.772-5.896	0.144			
FN1						
High vs. Low	3.801	3.801-7.289	**<0.001**	3.351	1.691-6.640	**0.001**

Bold values mean p < 0.05.

## Discussion

We thoroughly investigated the expression of FN1 in GC and its clinical relevance. We discovered that elevated FN1 expression was associated with a poor outcome in GC. Furthermore, our data also suggest that FN1 expression is closely related to the polarization of different immune cells, immune checkpoints, and macrophages in GC. Thus, our study reveals new insights into understanding key functions of FN1, which may be a prognostic biomarker associated with immune infiltration of GC.

FN1 is a ubiquitous ECM protein that has been implicated in a variety of key biological processes such as wound healing and embryonic morphogenesis, as well as cell adhesion and migration regulation (21–24). FN1 has been implicated in a variety of pathological processes, including cancer, infections, and rheumatoid arthritis (13, 14, 25, 26). Numerous studies have shown that FN1 in oral squamous cell carcinoma (27), ovarian carcinoma (28), nasopharyngeal carcinoma (29) is differentially expressed and demonstrates distinct functions in cancer proliferation, migration, and invasion (10, 30). Meanwhile, FN1 has been connected to cancer patients’ prognosis and treatment responsiveness (15, 31). However, the role and molecular processes of FN1 in GC are still poorly understood.

Therefore, we firstly assessed the expression of FN1 in GC through databases such as GEPIA, TIMER, and TCGA. We found aberrant expression of FN1 between cancerous and paracancerous tissues in various malignancies. Furthermore, FN1 was significantly elevated in GC samples compared with paracancerous samples. These results are consistent with those of TCGA database. Furthermore, to confirm whether FN1 can be used as a prognostic biomarker, we analyzed the correlation between FN1 expression and OS, PPS, and FP in the GC cohort using the KM plotter database. Notably, analysis of this database showed that poorer OS, PPS, and FP were associated with higher FN1 expression. Upregulated FN1 expression in T stage was significantly associated with worse prognosis in GC. The expression of FN1 and the genes closely related to its expression have a higher risk of death. Taken together, these observations support the hypothesis that FN1 is a prognostic biomarker for GC.

Furthermore, this study discovered that FN1 was directly related to the degree of immune infiltration in GC. In the tumor microenvironment, immune cell infiltration has been indicated to play a crucial role in cancer process (32, 33). The expression of FN1 in tumor invasion and migration is needed. However, whether the expression of FN1 is related to immune infiltration in GC remains ambiguous. Therefore, we investigated the association between FN1 expression and the degree of immune infiltration in GC. Our study shows that FN1 expression is closely related to tumor-infiltrating immune cells including macrophages, NK cells, Treg cells, CD8+ T cells, and DCs. At the same time, we have studied that the expression of FN1 is associated with immune checkpoints as well as markers of macrophages. Macrophages are divided into M1 and M2, and certain subsets of M2 macrophages are also involved in promoting tumor development (34, 35). Meanwhile, we discovered that FN1 expression was positively connected with CD163 by IHC, and that high FN1 expression was associated with worse OS and DFS. These data imply that FN1 may play a major role in controlling TAM polarization.

However, our study has certain limitations. First, most of our data are based on online platform databases, which are constantly being updated and expanded; therefore, research results may be affected. Second, the function of FN1 in GC and its underlying mechanisms in GC immunity were not experimentally investigated in our analysis. However, in the future, we will place a larger focus on the whole baseline information of patients, and studies will be conducted to further establish the predictive validity.

## Conclusions

Upregulated FN1 expression is closely associated with poor prognosis and enhanced degree of immune infiltration, including the expression of macrophages in GC, especially M2-type macrophages. Therefore, the present study suggests that FN1 serves as a prognostic biomarker that may highlight its novel potential function in regulating immune cell infiltration in GC patients.

## Data availability statement

The original contributions presented in the study are included in the article/**Supplementary Material**. Further inquiries can be directed to the corresponding authors.

## Ethics statement

The studies involving human participants were reviewed and approved by the ethics committee of the Seventh Affiliated Hospital of Sun Yat-sen University, Shenzhen, Guangdong, China. Informed consent was obtained from all subjects involved in the study. The patients/participants provided their written informed consent to participate in this study.

## Author contributions

YH and CZ designed and supervised the study. HW and JZ carried out the bioinformatics analysis, HW, JZ, HL performed the experiments, and analyzed the data. HW, JZ and HL wrote the paper. SC, SL, HY, YH and CZ edited the manuscript and provided critical comments. All authors contributed to the article and approved the submitted version.

## Funding

This study was funded by grants from the Guangdong Province Science and Technology Plan Projects (2014A020212693), “3&3” Project of The First Affiliated Hospital of Sun Yat-Sen University (YH), and Sanming Project of Medical in Shenzhen (SZSM201911010). All these study sponsors have no roles in the study design, in the analysis, and interpretation of data.

## Conflict of interest

The authors declare that the research was conducted in the absence of any commercial or financial relationships that could be construed as a potential conflict of interest.

## Publisher’s note

All claims expressed in this article are solely those of the authors and do not necessarily represent those of their affiliated organizations, or those of the publisher, the editors and the reviewers. Any product that may be evaluated in this article, or claim that may be made by its manufacturer, is not guaranteed or endorsed by the publisher.

## Footnotes

1
https://timer.cistrome.org/

2
http://kmplot.com/analysis/

3
http://www.linkedomics.org/login.php

4
http://gepia.cancer-pku.cn/index.html

5
https://www.cbioportal.org/

6
https://www.cancer.gov/tcga/

## References

[1] Sung H Ferlay J Siegel RL Laversanne M Soerjomataram I Jemal A. Global cancer statistics 2020: GLOBOCAN estimates of incidence and mortality worldwide for 36 cancers in 185 countries. CA Cancer J Clin (2021) 71(3):209–49. doi: 10.3322/caac.21660 33538338

[2] Dolcetti R De Re V Canzonieri V. Immunotherapy for gastric cancer: Time for a personalized approach? Int J Mol Sci (2018) 19(6):1602. doi: 10.3390/ijms19061602 PMC6032163 29844297

[3] Charalampakis N Economopoulou P Kotsantis I Tolia M Schizas D Liakakos T. Medical management of gastric cancer: a 2017 update. Cancer Med (2018) 7(1):123–33. doi: 10.1002/cam4.1274 PMC5773977 29239137

[4] Kwak Y Seo AN Lee HE Lee HS. Tumor immune response and immunotherapy in gastric cancer. J Pathol Transl Med (2020) 54:20–33. doi: 10.4132/jptm.2019.10.08 31674166 PMC6986974

[5] Quail DF Joyce JA. Microenvironmental regulation of tumor progression and metastasis. Nat Med (2013) 19:1423–37. doi: 10.1038/nm.3394 PMC3954707 24202395

[6] ffrench-Constant C. Alternative splicing of fibronectin–many different proteins but few different functions. Exp Cell Res (1995) 221:261–71. doi: 10.1006/excr.1995.1374 7493623

[7] Kraft S Klemis V Sens C Lenhard T Jacobi C Samstag Y. Identification and characterization of a unique role for EDB fibronectin in phagocytosis. J Mol Med (Berl) (2016) 94(5):567–81. doi: 10.1007/s00109-015-1373-0 PMC4856727 26637426

[8] Alexi X Berditchevski F Odintsova E. The effect of cell-ECM adhesion on signalling *via* the ErbB family of growth factor receptors. Biochem Soc Trans (2011) 39:568–73. doi: 10.1042/BST0390568 21428941

[9] Xia S Wang C Postma EL Yang Y Ni X Zhan W. Fibronectin 1 promotes migration and invasion of papillary thyroid cancer and predicts papillary thyroid cancer lymph node metastasis. Oncol Targets Ther (2017) 10:1743–55. doi: 10.2147/OTT.S122009 PMC5370387 28367057

[10] Xie Y Liu C Qin Y Chen J Fang J. Knockdown of IRE1ɑ suppresses metastatic potential of colon cancer cells through inhibiting FN1-Src/FAK-GTPases signaling. Int J Biochem Cell Biol (2019) 114:105572. doi: 10.1016/j.biocel.2019.105572 31326465

[11] Steffens S Schrader AJ Vetter G Eggers H Blasig H Becker J. Fibronectin 1 protein expression in clear cell renal cell carcinoma. Oncol Lett (2012) 3(4):787–90. doi: 10.3892/ol.2012.566 PMC3362387 22740994

[12] Efthymiou G Saint A Ruff M Rekad Z Ciais D Van Obberghen-Schilling E. Shaping up the tumor microenvironment with cellular fibronectin. Front Oncol (2020) 10:641. doi: 10.3389/fonc.2020.00641 32426283 PMC7203475

[13] Lin T-C Yang CH Cheng LH Chang WT Lin YR Cheng HC. Fibronectin in cancer: Friend or foe. Cells (2019) 9(1):27. doi: 10.3390/cells9010027 PMC7016990 31861892

[14] Speziale P Arciola CR Pietrocola G. Fibronectin and its role in human infective diseases. Cells (2019) 8(12):1516. doi: 10.3390/cells8121516 PMC6952806 31779172

[15] Ye Y Zhang R Feng H. Fibronectin promotes tumor cells growth and drugs resistance through a CDC42-YAP-dependent signaling pathway in colorectal cancer. Cell Biol Int (2020) 44:1840–9. doi: 10.1002/cbin.11390 32437085

[16] Peranzoni E Lemoine J Vimeux L Feuillet V Barrin S Kantari-Mimoun C. Macrophages impede CD8 T cells from reaching tumor cells and limit the efficacy of anti-PD-1 treatment. Proc Natl Acad Sci U S A (2018) 115(17):E4041–50. doi: 10.1073/pnas.1720948115 PMC5924916 29632196

[17] Peranzoni E Rivas-Caicedo A Bougherara H Salmon H Donnadieu E. Positive and negative influence of the matrix architecture on antitumor immune surveillance. Cell Mol Life Sci (2013) 70:4431–48. doi: 10.1007/s00018-013-1339-8 PMC11113382 23649148

[18] Salmon H Franciszkiewicz K Damotte D Dieu-Nosjean MC Validire P Trautmann A. Matrix architecture defines the preferential localization and migration of T cells into the stroma of human lung tumors. J Clin Invest (2012) 122(3):899–910. doi: 10.1172/JCI45817 22293174 PMC3287213

[19] Liguori M Solinas G Germano G Mantovani A Allavena P. Tumor-associated macrophages as incessant builders and destroyers of the cancer stroma. Cancers (Basel) (2011) 3:3740–61. doi: 10.3390/cancers3043740 PMC3763394 24213109

[20] Afik R Zigmond E Vugman M Klepfish M Shimshoni E Pasmanik-Chor M. Tumor macrophages are pivotal constructors of tumor collagenous matrix. J Exp Med (2016) 213(11):2315–31. doi: 10.1084/jem.20151193 PMC5068227 27697834

[21] Park J Schwarzbauer JE. Mammary epithelial cell interactions with fibronectin stimulate epithelial-mesenchymal transition. Oncogene (2014) 33:1649–57. doi: 10.1038/onc.2013.118 PMC3934944 23624917

[22] Wang Y Ni H. Fibronectin maintains the balance between hemostasis and thrombosis. Cell Mol Life Sci (2016) 73:3265–77. doi: 10.1007/s00018-016-2225-y PMC11108312 27098513

[23] Maione AG Smith A Kashpur O Yanez V Knight E Mooney DJ. Altered ECM deposition by diabetic foot ulcer-derived fibroblasts implicates fibronectin in chronic wound repair. Wound Repair Regen (2016) 24(4):630–43. doi: 10.1111/wrr.12437 PMC5500637 27102877

[24] Wang J Li R Li M Wang C. Fibronectin and colorectal cancer: signaling pathways and clinical implications. J Recept Signal Transduction Res (2021) 41:313–20. doi: 10.1080/10799893.2020.1817074 32900261

[25] Calderone RA Scheld WM. Role of fibronectin in the pathogenesis of candidal infections. Rev Infect Dis (1987) 9 (Suppl 4):S400–3. doi: 10.1093/clinids/9.Supplement_4.S400 3326135

[26] Elices MJ Tsai V Strahl D Goel AS Tollefson V Arrhenius T. Expression and functional significance of alternatively spliced CS1 fibronectin in rheumatoid arthritis microvasculature. J Clin Invest (1994) 93(1):405–16. doi: 10.1172/JCI116975 PMC293796 8282813

[27] Yen CY Huang CY Hou MF Yang YH Chang CH Huang HW. Evaluating the performance of fibronectin 1 (FN1), integrin α4β1 (ITGA4), syndecan-2 (SDC2), and glycoprotein CD44 as the potential biomarkers of oral squamous cell carcinoma (OSCC). Biomarkers (2013) 18(1):63–72. doi: 10.3109/1354750X.2012.737025 23116545

[28] Lou X Han X Jin C Tian W Yu W Ding D. SOX2 targets fibronectin 1 to promote cell migration and invasion in ovarian cancer: new molecular leads for therapeutic intervention. OMICS (2013) 17(10):510–8. doi: 10.1089/omi.2013.0058 PMC3783972 23895273

[29] Ma L-J Lee SW Lin LC Chen TJ Chang IW Hsu HP. Fibronectin overexpression is associated with latent membrane protein 1 expression and has independent prognostic value for nasopharyngeal carcinoma. Tumour Biol (2014) 35(2):1703–12. doi: 10.1007/s13277-013-1235-8 24081675

[30] Cai X Liu C Zhang TN Zhu YW Dong X Xue P. Down-regulation of FN1 inhibits colorectal carcinogenesis by suppressing proliferation, migration, and invasion. J Cell Biochem (2018) 119(6):4717–28. doi: 10.1002/jcb.26651 29274284

[31] Sun Y Zhao C Ye Y Wang Z He Y Li Y. High expression of fibronectin 1 indicates poor prognosis in gastric cancer. Oncol Lett (2020) 19(1):93–102. doi: 10.3892/ol.2019.11088 31897119 PMC6923922

[32] Arneth B. Tumor microenvironment. Med (Kaunas) (2019) 56(1):15. doi: 10.3390/medicina56010015 PMC7023392 31906017

[33] Wang M Zhao J Zhang L Wei F Lian Y Wu Y. Role of tumor microenvironment in tumorigenesis. J Cancer (2017) 8(5):761–73. doi: 10.7150/jca.17648 PMC5381164 28382138

[34] Atri C Guerfali FZ Laouini D. Role of human macrophage polarization in inflammation during infectious diseases. Int J Mol Sci (2018) 19(6):1801. doi: 10.3390/ijms19061801 PMC6032107 29921749

[35] Funes SC Rios M Escobar-Vera J Kalergis AM. Implications of macrophage polarization in autoimmunity. Immunology (2018) 154:186–95. doi: 10.1111/imm.12910 PMC5980179 29455468

