# Supplementary material for: FN1 is a prognostic biomarker and correlated with immune infiltrates in gastric cancers
Source: Front Oncol. 2022 Aug 23;12:918719. doi: 10.3389/fonc.2022.918719 (PMC9445423; doi:10.3389/fonc.2022.918719)
Supplement: Supplementary file 1 [file Table_1.docx]

Supplementary Material

**Supplementary Table 1.** Somatic copy number variation (CNV) of FN1 at the level of immune infiltration

| cancer | variable | cna_level | p |
| --- | --- | --- | --- |
| STAD | B Cell | Deep Deletion | 1 |
| STAD | B Cell | Arm-level Deletion | 0.164118997 |
| STAD | B Cell | Diploid/Normal | 1 |
| STAD | B Cell | Arm-level Gain | 0.221281334 |
| STAD | B Cell | High Amplication | 0.36826804 |
| STAD | CD8+ T Cell | Deep Deletion | 1 |
| STAD | CD8+ T Cell | Arm-level Deletion | 0.000716809 |
| STAD | CD8+ T Cell | Diploid/Normal | 1 |
| STAD | CD8+ T Cell | Arm-level Gain | 0.006842919 |
| STAD | CD8+ T Cell | High Amplication | 0.456325904 |
| STAD | CD4+ T Cell | Deep Deletion | 1 |
| STAD | CD4+ T Cell | Arm-level Deletion | 0.001271971 |
| STAD | CD4+ T Cell | Diploid/Normal | 1 |
| STAD | CD4+ T Cell | Arm-level Gain | 0.094904481 |
| STAD | CD4+ T Cell | High Amplication | 0.697137865 |
| STAD | Macrophage | Deep Deletion | 1 |
| STAD | Macrophage | Arm-level Deletion | 0.2442611 |
| STAD | Macrophage | Diploid/Normal | 1 |
| STAD | Macrophage | Arm-level Gain | 0.000219064 |
| STAD | Macrophage | High Amplication | 0.789861777 |
| STAD | Neutrophil | Deep Deletion | 1 |
| STAD | Neutrophil | Arm-level Deletion | 1.14E-06 |
| STAD | Neutrophil | Diploid/Normal | 1 |
| STAD | Neutrophil | Arm-level Gain | 0.015376911 |
| STAD | Neutrophil | High Amplication | 0.230777214 |
| STAD | Dendritic Cell | Deep Deletion | 1 |
| STAD | Dendritic Cell | Arm-level Deletion | 3.07E-05 |
| STAD | Dendritic Cell | Diploid/Normal | 1 |
| STAD | Dendritic Cell | Arm-level Gain | 0.002112501 |
| STAD | Dendritic Cell | High Amplication | 0.649705976 |

**Supplementary Table 2** Relationship between FN1 expression and clinicopathological parameters in patients with gastric cancer.

| Characteristic | FN1 | | |  |
| --- | --- | --- | --- | --- |
|  | Low (%) | High (%) | P |  |
| Age (years) |  |  | 0.531 |  |
| ＜60 | 35(34.3) | 67(65.7) |  |  |
| ≥60 | 19(39.6) | 29(60.4) |  |  |
| Gender |  |  | 0.89 |  |
| Male | 32(35.6) | 58(64.4) |  |  |
| Female | 22(36.7) | 38(63.3) |  |  |
| Tumor size |  |  | **<0.001** |  |
| ≤5 cm | 50(46.7) | 57(53.3) |  |  |
| ＞5 cm | 4(9.3) | 39(90.7) |  |  |
| Borrmann type |  |  | **<0.001** |  |
| I-II | 6(22.2) | 21(77.8) |  |  |
| III-IV | 11(13.3) | 72(86.7) |  |  |
| Differentiation |  |  | 0.259 |  |
| poor | 3(60) | 2(40) |  |  |
| moderate | 9(47.4) | 10(52.6) |  |  |
| Well | 42(33.3) | 84(66.7) |  |  |
| pTNM stage |  |  | **<0.001** |  |
| I | 43(100) | 0(0) |  |  |
| II | 11(21.2) | 41(78.8) |  |  |
| III | 0(0) | 55(100) |  |  |
| Depth of invasion |  |  | **<0.001** |  |
| T1/2 | 45(88.2) | 6(11.8) |  |  |
| T3/4 | 9(9.1) | 90(90.9) |  |  |
| Lymph node metastasis |  |  | **<0.001** |  |
| N0 | 44(62.9) | 26 (37.1) |  |  |
| N+ | 10(12.5) | 70 (87.5) |  |  |
| LVI |  |  | 0.088 |  |
| Yes | 0(0) | 6(100) |  |  |
| No | 54(37.5) | 90(62.5) |  |  |
|  |  |  |  |  |

**Supplementary Table 3** Univariate and multivariate COX risk regression analysis of disease-free survival of patients with gastric cancer

| Variable | Univariate | | |  | Multivariate | | |
| --- | --- | --- | --- | --- | --- | --- | --- |
|  | HR | 95%CI | P |  | HR | 95%CI | P |
| Age (years) |  |  |  |  |  |  |  |
| ≥60 vs.＜60 | 1.562 | 0.951-2.566 | 0.078 |  |  |  |  |
| Gender |  |  |  |  |  |  |  |
| Male vs. Female | 1.602 | 0984-2.607 | 0.058 |  |  |  |  |
| Tumor size |  |  |  |  |  |  |  |
| ＞5 cm vs. ≤5 cm | 3.716 | 2.250-6.137 | **<0.001** |  | 1.885 | 1.123-3.164 | **0.016** |
| Borrmann type |  |  |  |  |  |  |  |
| III-IV vs. I-II | 2.698 | 1.645-4.424 | **<0.001** |  |  |  |  |
| Differentiation |  |  |  |  |  |  |  |
| Poor vs.  Well+ moderate | 2.749 | 0.381-19.822 | 0.316 |  |  |  |  |
| Depth of invasion |  |  |  |  |  |  |  |
| T3-4 vs. T1-2 | 5.025 | 2.166-11.655 | **<0.001** |  |  |  |  |
| Lymph node metastasis |  |  |  |  |  |  |  |
| N+ vs. N0 | 2.796 | 1.636-4.779 | **<0.001** |  |  |  |  |
| LVI |  |  |  |  |  |  |  |
| Present vs. none | 2.17 | 0.787-5.98 | 0.134 |  |  |  |  |
| FN1 |  |  |  |  |  |  |  |
| High vs. Low | 3.699 | 1.93-7.088 | **<0.001** |  | 3.312 | 1.671-6.564 | **0.001** |
